# Thiazolidinediones inhibit airway smooth muscle release of the chemokine CXCL10: in vitro comparison with current asthma therapies

**DOI:** 10.1186/1465-9921-13-90

**Published:** 2012-10-04

**Authors:** Petra Seidel, Hatem Alkhouri, Daniel J Lalor, Janette K Burgess, Carol L Armour, J Margaret Hughes

**Affiliations:** 1 Respiratory Research Group, Faculty of Pharmacy, The University of Sydney, A15, Science Rd, Sydney, NSW 2006, Australia; 2 Respiratory Research Group, Discipline of Pharmacology, The University of Sydney, Sydney, Australia; 3 Woolcock Institute of Medical Research, Sydney, NSW Australia; 4 Current affiliation: Discipline of Pharmacology, The University of Sydney and Woolcock Institute of Medical Research, Sydney, NSW Australia

**Keywords:** Mast cell chemoattractant, Glucocorticoids, Long-acting β2-agonists, Salmeterol, Fluticasone, Ciglitazone, Rosiglitazone

## Abstract

**Background:**

Activated mast cells are present within airway smooth muscle (ASM) bundles in eosinophilic asthma. ASM production of the chemokine CXCL10 plays a role in their recruitment. Thus the effects of glucocorticoids (fluticasone, budesonide), long-acting β2-agonists (salmeterol, formoterol) and thiazolidinediones (ciglitazone, rosiglitazone) on CXCL10 production by ASM cells (ASMC) from people with and without asthma were investigated in vitro.

**Methods:**

Confluent serum-deprived cells were treated with the agents before and during cytokine stimulation for 0-24 h. CXCL10 protein/mRNA, IκB-α levels and p65 activity were measured using ELISA, RT PCR, immunoblotting and p65 activity assays respectively. Data were analysed using ANOVA followed by Fisher’s post-hoc test.

**Results:**

Fluticasone and/or salmeterol at 1 and 100 nM inhibited CXCL10 release induced by IL-1β and TNF-α, but not IFNγ or all three cytokines (cytomix). The latter was also not affected by budesonide and formoterol. In asthmatic ASMC low salmeterol, but not formoterol, concentrations increased cytomix-induced CXCL10 release and at 0.01 nM enhanced NF-κB activity. Salmeterol 0.1nM together with fluticasone 0.1 and 10 nM still increased CXCL10 release. The thiazolidinediones ciglitazone and rosiglitazone (at 25 and 100 μM) inhibited cytomix-induced CXCL10 release but these inhibitory effects were not prevented by the PPAR-g antagonist GW9662. Ciglitazone did not affect early NF-κB activity and CXCL10 mRNA production.

**Conclusions:**

Thus the thiazolidinediones inhibited asthmatic ASMC CXCL10 release under conditions when common asthma therapies were ineffective or enhanced it. They may provide an alternative strategy to reduce mast cell-ASM interactions and restore normal airway physiology in asthma.

## Background

Asthma is a disease of the airways characterized by airway obstruction and airway hyperresponsiveness. The airways of people with asthma are inflamed and undergo continuous remodelling resulting in thicker airway walls leading to airflow limitation [1]. The interaction between airway smooth muscle cells (ASMC) and activated mast cells may be crucial in the pathogenesis of asthma [2]. The number of mast cells is increased in the airway smooth muscle (ASM) layer of asthmatic patients when compared with non-asthmatic patients and the increase in activated mast cell numbers in asthmatic ASM correlates with the degree of airway hyper-responsiveness [3,4].

The question of how mast cells accumulate in the ASM is an area of active research. A variety of ASMC-derived chemotactic and survival factors for mast cells have been identified, including transforming growth factor (TGF)-β, CX3CL1 (fractalkine), stem cell factor and CXCL10 (interferon inducible protein of 10 kDa (IP-10)) [5-8]. Previously we reported that ASMC induce human lung mast cell migration via CXCR3 activation on the mast cells [8]. CXCR3 is the most abundantly expressed chemokine receptor on mast cells found within the ASM bundle and CXCL10, a potent mast cell chemoattractant and CXCR3 receptor ligand, is released from ASMC. Most importantly, ASM in biopsies from asthmatics express CXCL10 more often than controls [8] and it is produced faster by asthmatic compared with non-asthmatic ASMC in vitro [9]. Our previous findings are consistent with CXCL10 playing an important role in mast cell accumulation in the ASM. Thus how ASM CXCL10 production can be inhibited most effectively is of considerable interest.

To date the combination of inhaled glucocorticoids (GC) and long-acting β-2 agonists (LABA) remains the most effective treatment for asthma. Researchers have shown in numerous in vitro studies that GC alone and in combination with LABA reduce pro-inflammatory cytokine secretion from non-asthmatic ASMC, including the chemokines CXCL8 (IL-8) [10], CCL11 (eotaxin) [11] and CCL5 (RANTES) [12]. However other chemokines, particularly those induced by interferon(IFN)γ, are resistant [13] and there are some asthma patients where GC therapy is insufficient to control asthma symptoms [14]. Therefore, it has been proposed that the addition of other anti-inflammatory drugs such as peroxisome proliferator-activated receptor (PPAR)γ agonists could help to control asthma in difficult to treat patients [15].

PPARγ agonists such as the thiazolidinediones are currently licensed for the treatment of diabetes mellitus and are thought to exert their effects mainly through activation of the PPARγ receptor. ASM PPARγ expression is increased in bronchial biopsies from people with asthma [16] and thiazolidinediones reduce airway hyperreponsiveness in animal models of allergic airways disease [17,18]. However, there is evidence that PPARγ agonists can also modulate the function of other pro-inflammatory transcription factors such as nuclear factor(NF)-κB [19,20]. Anti-inflammatory effects of PPARγ agonists have been described *in vivo* in animal models [17,21,22], as well as *in vitro* on ASMC [15,23]. It has been hypothesized that adding this group of drugs could therefore be beneficial in the therapy of asthma, particularly when there is not an adequate response to current asthma medications [15].

The aims of this study were to investigate the individual and combined effects of the GC fluticasone with the LABA salmeterol or the thiazolidinedione ciglitazone, on cytokine-induced CXCL10 release by ASMC from people with and without asthma.

## Methods

### Materials

Salmeterol and fluticasone propionate (Glaxo Wellcome R&D, Hertfordshire, UK and Sigma-Aldrich, Australia), budesonide and formoterol (AstraZeneca R&D, Lund, Sweden) were freshly prepared in dimethyl sulfoxide (DMSO) prior to use. Stocks of other agents were also prepared in DMSO and aliquots stored at −20°C. The thiazolidinediones ciglitazone (Sigma-Aldrich, Australia) and rosiglitazone (kind gift of Professor David Hibbs) and the β2-adrenoceptor antagonist butoxamine (Sigma-Aldrich, Australia) were prepared as 10 mM stocks. The irreversible PPARγ antagonist GW-9662 (Cayman, MI, USA) was prepared as a 100 mM stock in DMSO. Recombinant human interleukin(IL)-1β, tumour necrosis factor(TNF)-α (R&D Systems, Minneapolis, MN, USA) and recombinant human IFNγ (BD Biosciences) were reconstituted in sterile PBS containing 0.1% bovine serum albumin (BSA) at 10 μg/ml and aliquots stored at −20°C and −80°C respectively. All agents were diluted to the appropriate working concentration in the medium used to serum-deprive the cells.

### Airway smooth muscle cell culture

Samples of resected lung and bronchial biopsies were obtained and used with the ethical approval of the Sydney South West Area Health Service and the Australian Red Cross. Approval for this study was provided by the Human Ethics Committee of The University of Sydney. The people without asthma had a mean age of 59 ± 15.3 (SD) years (n=21), whereas those with asthma had a mean age of 33.5 ± 13.6 years (n=18) and a positive bronchial challenge to mannitol [24] or methacholine [25] and symptoms in the last 12 months.

Smooth-muscle bundles were dissected from the bronchial tissue and grown as explants as described previously [8,26] in DMEM supplemented with 10% FBS, 100 units/mL penicillin G, 100 μg/mL streptomycin sulphate, 25 μg/mL amphotericin B, 4 mM L-glutamine and 20 mM of HEPES growth medium pH 7.4, in a humidified 5% carbon dioxide in air atmosphere at 37°C. ASMC were identified by their morphology, positive immunohistochemical staining for α-smooth muscle actin and calponin and their typical ASMC “hill and valley” pattern of growth. From passages 4–7 the cells were harvested with trypsin-EDTA, washed and plated at a density of 1 × 10^4^ cells/cm^2^ in growth medium for all experiments, except where nuclear extracts were generated, when cells were plated at a density of 2.5 × 10^4^ cells/cm^2^. Before and at the end of any treatment period the morphology and viability of the plated cells were checked using phase contrast microscopy.

### Drug treatment, cell stimulation and CXCL10 production

The effects of GC and LABA on cytokine induced CXCL10 release were determined on ASMC from asthmatic and non-asthmatic donors. The cells were grown for a week and then serum starved using DMEM supplemented with antibiotics, 4 mM L-glutamine, 20 mM HEPES (pH 7.4) and 0.1% BSA. After 48 h, ASMC were treated with salmeterol (0.1-100 nM) and/or fluticasone (0.1-100 nM) or budesonide (0.1-100 nM) and/or formoterol (0.1-100 nM) for 1 h prior to and during stimulation with either IL-1β, TNF-α and IFNγ alone, or in combination (cytomix), at a final cytokine concentration of 10 ng/ml each. In studies to determine β2-adrenoceptor involvement, asthmatic ASMC were treated with the selective β2-adrenoceptor antagonist butoxamine at a final concentration of 30 μM [27] 1 h prior to and during the salmeterol treatment and cytomix stimulation of the cells.

To investigate the effects of the thiazolidinediones, ciglitazone and rosiglitazone, on cytokine induced CXCL10 release, ASMC were treated with either agent at 25 μM or 100 μM [28] for 0.5 h before and during cytokine stimulation of the cells as above. The PPARγ antagonist GW-9662 (10 μM) [29] was also added to specified wells 0.5 h prior to treating the cells with ciglitazone or rosiglitazone. To determine whether fluticasone (0.1-100 nM) or salmeterol (0.1-100 nM) in combination with ciglitazone (25 μM or 100 μM) caused additional effects, asthmatic and non-asthmatic ASMC were treated with both drugs 1 h prior to cytomix stimulation as described above. All experiments had untreated and vehicle controls. After a further 24 h incubation the culture medium from each well (supernatant) was collected, stored at −20°C and later analysed using CXCL10 Duo-set ELISA kits (R&D Systems) according to the manufacturer’s instructions.

The effects of ciglitazone on ASMC CXCL10 mRNA levels induced during 3 h stimulation with cytomix were also determined as described previously [9]. Total RNA was extracted from cell lysates using a NucleoSpin RNA 11 kit and protocol (Macherey-Nagel GmbH & Co, Duren Germany). Reverse transcription of 1 μg of extracted RNA was performed using the RevertAid First Strand cDNA Synthesis Kit and protocol for the use of random hexamer primers (Fermentas Life Sciences, Hanover, MD). The resulting cDNA (2 μl) was amplified using standard conditions and the CXCL10 primer sets (Hs00171042_m1) multiplexed with the eukaryotic 18S rRNA endogenous control probe (Applied Biosystems, Melbourne Australia). Results for CXCL10 were normalized against those for 18S and finally expressed as fold change over the vehicle control.

### NF-B p65 DNA binding activity

To investigate the effects of salmeterol (0.001-0.01 nM) and ciglitazone (25 μM or 100 μM) on NF-κB p65 transcription factor DNA binding activity, ASMC from asthmatic and non-asthmatic donors were treated with the drugs and then stimulated with cytomix as before for 15 and 30 min. Nuclear extracts were generated using a NE-PER nuclear and cytoplasmic extraction kit (Pierce Biotechnology, Rockford, IL, USA) and nuclear NF-κB p65 DNA binding activities measured using NF-kappa B (p65) transcription factor activity assays (Cayman, Ann Arbor, MI, USA) according to the manufacturers’ protocols.

### IB- degradation

Total cell extracts were generated from ASMC treated as described above, to examine IκB-α degradation. After SDS-PAGE of these extracts, membranes were incubated with the primary antibodies anti-IκBα (C21, 1:1000) and anti-α-tubulin (DM1A, 1:1000, both Santa Cruz Biotechnology, Santa Cruz, CA). The primary antibodies were then detected using horseradish peroxidase-conjugated IgG antibodies (1:2000, anti-rabbit IgG or anti-mouse IgG, both Cell Signaling Technology, Beverly, MA) and visualized using enhanced chemiluminescence (PerkinElmer, Wellesley, MA).

### Data analysis

CXCL10 concentrations (pg/ml, ELISA) or optical densities (nm, NF-κB activity assay) from replicate treatments were averaged and standardized to the specified controls. The mean ± SEM was then calculated for the ASMC from donors with or without asthma. Statistical analyses were performed on all data using Statview and significance (p<0.05) was determined by 1- or 2-way ANOVA followed by a Fisher post-hoc test.

## Results

### GC and LABA modulation of cytokine-induced CXCL10 release

**Figure 1:**
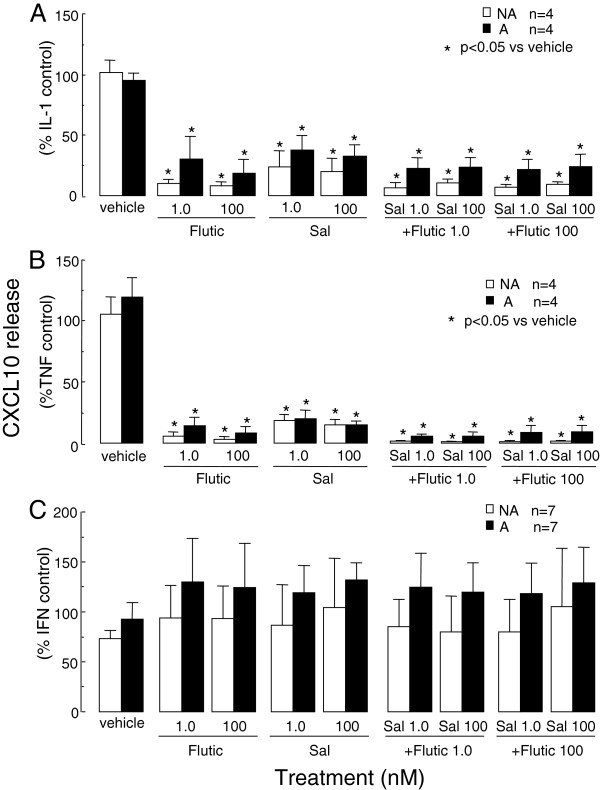
**The effects of fluticasone (Flutic) and salmeterol (Sal) on cytokine-induced CXCL10 release by asthmatic (A) and non-asthmatic (NA) ASMC. **Confluent serum-deprived ASMC were treated with fluticasone and/or salmeterol for 1 h prior to and during stimulation with the cytokines (each at 10 ng/ml) **A**) IL-1β, **B**) TNF-α or **C**) IFNγ and CXCL10 release after 24 h quantified by ELISA. Bars, mean ± SEM.

The effect of current asthma therapies on cytokine-induced CXCL10 release were stimulus specific. IL-1β- or TNF-α-induced CXCL10 production by asthmatic (2145 ± 691 and 13928 ± 4773 pg/ml respectively) and non-asthmatic (1565 ± 255 and 9043 ± 4140 pg/ml respectively) ASMC after 24 h of stimulation was significantly inhibited by salmeterol (1 and 100 nM) and fluticasone (1 and 100 nM). CXCL10 levels were similar to basal release (501 ± 254 and 406 ± 214 pg/ml respectively) when the drugs were used in combination (Figure 1 A and B). However the drugs, individually or in combination, failed to inhibit IFNγ-induced CXCL10 release by both asthmatic (15216 ± 5475 pg/ml) and non-asthmatic (6354 ± 2252 pg/ml) ASMC (Figure 1C).

**Figure 2:**
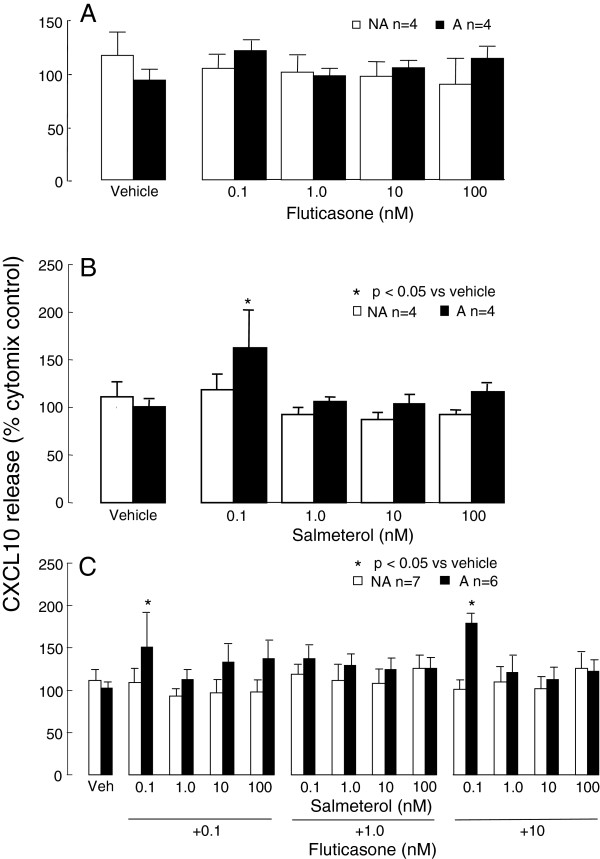
**The effects of fluticasone and salmeterol on asthmatic (A) and non-asthmatic (NA) ASMC CXCL10 release induced by cytomix. **Confluent serum-deprived ASMC were treated with **A**) fluticasone, **B**) salmeterol or **C**) both agents for 1 h prior to and during stimulation with cytomix (IL-1β, TNF-α and IFNγ combined, each at 10 ng/ml) and CXCL10 release after 24 h quantified by ELISA. Bars, mean ± SEM.

Individually (Figure 2A,B) or in combination (Figure 2C), did not inhibit cytomix-induced CXCL10 release from either asthmatic (65764 ± 9578 pg/ml) or non-asthmatic (51800 ± 6006 pg/ml) ASMC. Unexpectedly, the lowest concentration of salmeterol (0.1 nM) used in these experiments increased CXCL10 release to 163 ± 40% (p<0.05, n=6, Figure 2B) from asthmatic ASMC only. In addition, the lowest concentration of salmeterol (0.1 nM) in combination with fluticasone (0.1 and 10 nM) significantly increased cytomix-induced CXCL10 release from asthmatic but not from non-asthmatic, ASMC to 151 ± 39% and 180 ± 11% respectively (Figure 2C).

**Figure 3:**
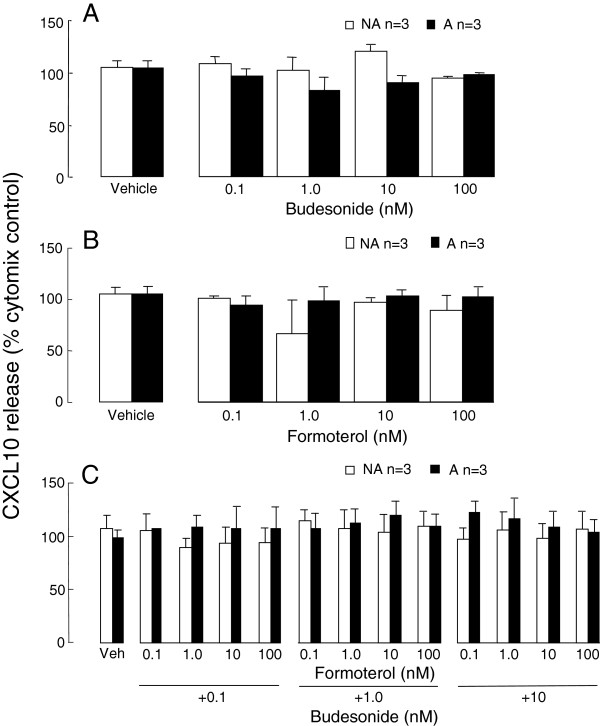
**The effects of budesonide and formoterol on asthmatic (A) and non-asthmatic (NA) ASMC CXCL10 release induced by cytomix. **Confluent serum-deprived ASMC were treated with **A**) budesonide, **B**) formoterol or **C**) both agents for 1 h prior to and during stimulation with cytomix (IL-1β, TNF-α and IFNγ combined, each at 10 ng/ml) and CXCL10 release after 24 h quantified by ELISA. Bars, mean ± SEM.

The effects of formoterol (0.1, 1, 10 and 100 nM) and budesonide (0.1, 1, 10 and 100 nM), by themselves or in combination, were also investigated in parallel with salmeterol and fluticasone in three of the same experiments and ASMC lines. In contrast to the salmeterol findings above, there were no significant changes in cytomix-induced CXCL10 production from either asthmatic or non-asthmatic ASMC with these agents (Figure 3A-C).

### 2-adrenoceptor involvement in the effects of salmeterol

**Figure 4:**
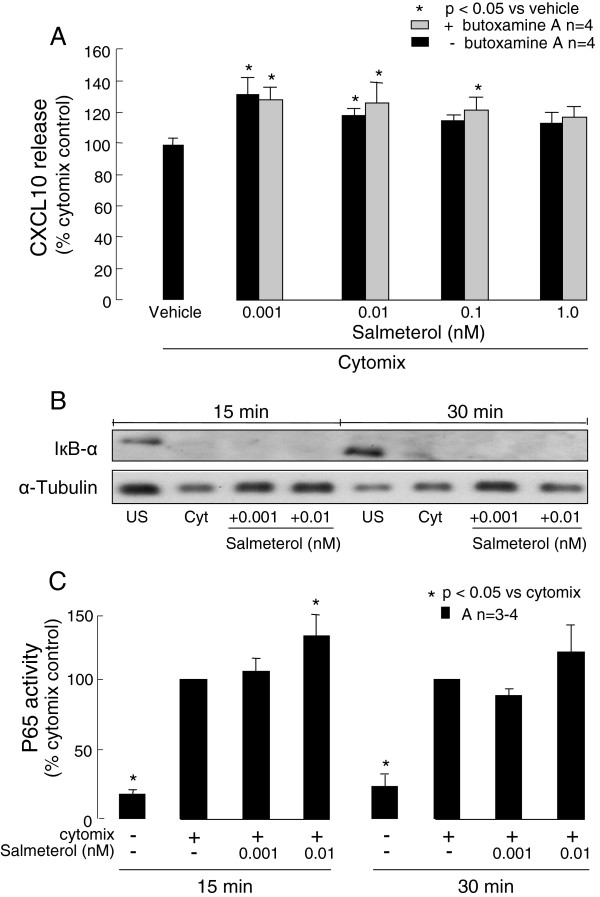
**β2-adrenoceptors and NF-κB involvement in the effects of salmeterol on asthmatic (A) ASMC. **The effects of lower salmeterol concentrations on **A**) CXCL10 release in the presence or absence of the selective β2-adrenoceptor antagonist butoxamine 30 μM or its vehicle, or **B**) IκB-α degradation (a representative immunoblot from 1 of 4 different ASMC examined) and **C**) NF-κB p65 DNA binding activity by confluent serum-deprived cells stimulated with cytomix for 24 h (A), or up to 30 min (B and C), were determined. Butoxamine was added to the cells 1 h before salmeterol. US, unstimulated; cyt, cytomix.

When lower concentrations of salmeterol, 0.01 and 0.001 nM, were used they also significantly increased cytomix-induced CXCL10 production by asthmatic ASMC to 118 ±4% and 127 ± 11% respectively (p<0.05, n=4, Figure 4A). Pre-treating the cells with the selective β2-adrenoceptor antagonist butoxamine (30 μM) did not reverse the salmeterol induced increases in CXCL10 release by the asthmatic cells (Figure 4A).

### NF-B involvement in the effects of salmeterol

As the transcription factor NF-κB is involved in ASMC CXCL10 production, the effects of low salmeterol concentrations on the NF-κB pathway in asthmatic ASMC were investigated. IκB-α remained fully degraded in the presence of salmeterol at the concentrations indicated at 15 and 30 min following cytomix stimulation (Figure 4B). Interestingly after 15 min of cell stimulation salmeterol, 0.01 nM but not 0.001 nM, significantly increased NF-κB p65 DNA binding activity by more than 20% (p<0.05, n=4, Figure 4C).

None of the above treatments affected the viability of the ASMC.

### The effects of the thiazolidinediones on cytokine induced CXCL10 release

**Figure 5:**
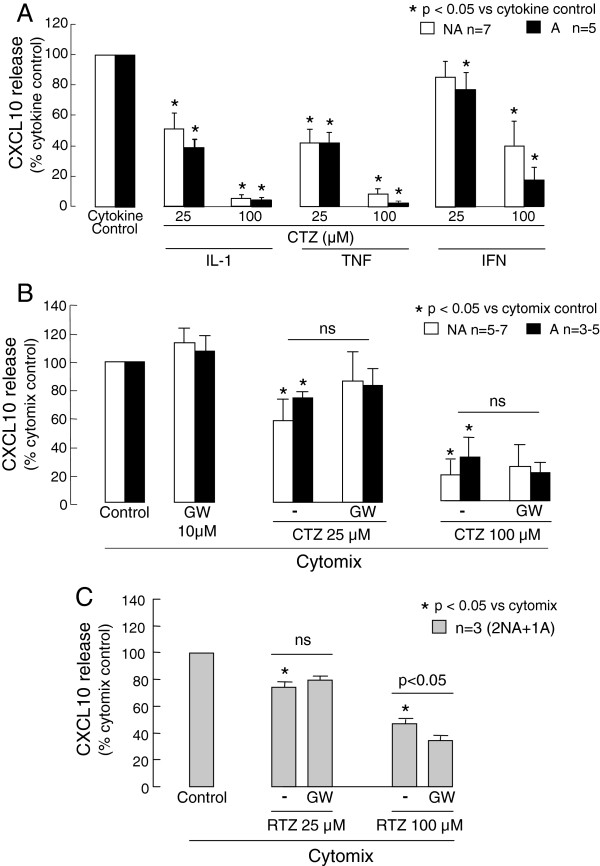
**The effects of ciglitazone (CTZ) and rosiglitazone (RTZ) on cytokine-induced CXCL10 release by asthmatic (A) and non-asthmatic (NA) ASMC. **Confluent serum-deprived ASMC were treated with vehicle or **A**) ciglitazone for 0.5 h and then stimulated with the cytokines (10 ng/ml each) IL-1β, TNF-α or IFNγ, or **B**) and **C**) the PPARγ antagonist GW9662 for 0.5 h prior to and during treatment with **B**) ciglitazone or **C**) rosiglitazone for 0.5 h followed by stimulation with cytomix (IL-1β, TNF-α and IFNγ combined, each at 10 ng/ml). CXCL10 release after 24 h cytokine stimulation was quantified.

Ciglitazone (25 and 100 μM) significantly reduced asthmatic and non-asthmatic ASMC CXCL10 release induced by the individual cytokines or cytomix (Figure 5A and B). However, it was less effective against IFNγ- or cytomix-induced CXCL10 release than IL-1β- or TNF-α-induced release. The PPARγ antagonist GW-9662 (10 μM) did not prevent the inhibitory effects of ciglitazone 25 and 100 μM on cytomix-induced CXCL10 release from either asthmatic or non-asthmatic ASMC (Figure 5B).

Rosiglitazone also significantly reduced cytomix-induced CXCL10 release by ASMC from one asthmatic and two non-asthmatic donors. As for ciglitazone, the PPARγ antagonist GW-9662 did not prevent the inhibitory effects of rosiglitazone on CXCL10 release (Figure 5C).

### The effects of ciglitazone on the activation of NF-B and CXCL10 gene expression

**Figure 6:**
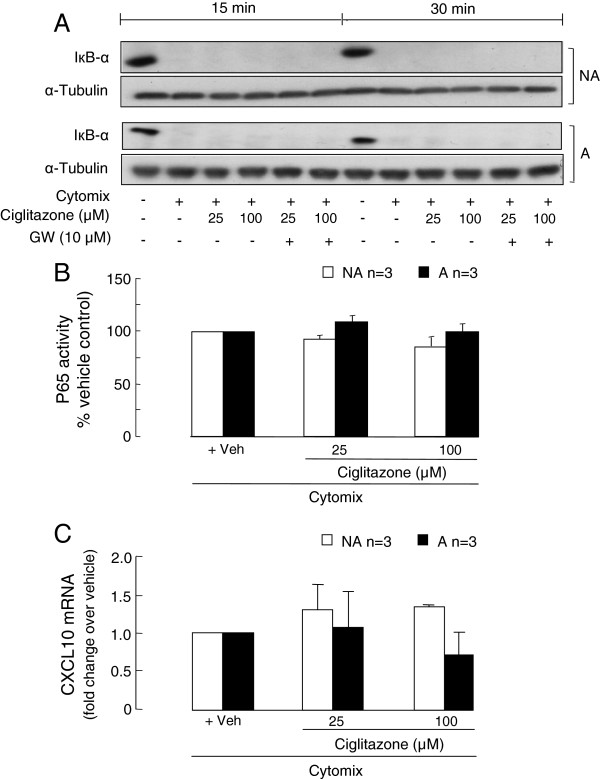
**The effects of ciglitazone on NF-κB activation and CXCL10 gene transcription. **Confluent serum-deprived asthmatic (**A**) and non-asthmatic (NA) ASMC were treated with the PPARγ antagonist GW-9662 and/or ciglitazone prior to and during stimulation with cytomix and **A**) IκB-α degradation, **B**) NF-κB p65 DNA binding activity and **C**) CXCL10 mRNA levels determined at 15 and 30 min (**A** and **B**) or 3 h using RT-PCR (**C**) respectively.

As PPARγ agonists can negatively interfere with the transcription factor NF-κB p65 activity, the effects of ciglitazone, with and without the antagonist GW-9662, on IκB-α degradation and NF-κB p65 activity were investigated. None of the ciglitazone concentrations used had an effect on IκB-α degradation or NF-κB activity, detected in whole cell lysates or nuclear extracts respectively, in the first 0.5 h after cytomix stimulation of the asthmatic and non-asthmatic ASMC (Figure 6A and B).

Given early signalling events leading to NF-κB-mediated gene transcription were not affected, we then determined the effect of ciglitazone on CXCL10 mRNA levels. Ciglitazone had no significant effects on CXCL10 mRNA levels in the asthmatic and non-asthmatic ASMC following 3 h cytomix stimulation (Figure 6C).

### The effects of asthma therapies in combination with ciglitazone

**Figure 7:**
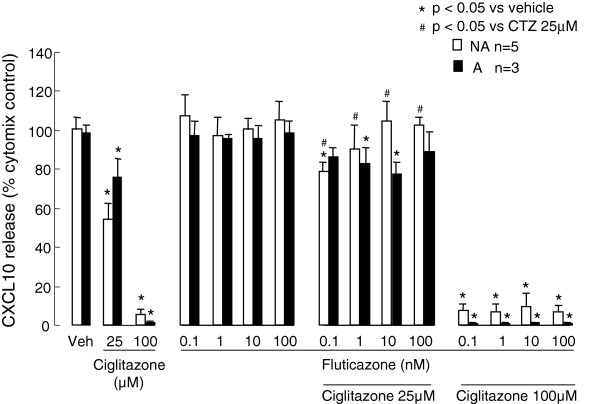
**The effects of ciglitazone and fluticasone on asthmatic (A) and non-asthmatic (NA) ASMC CXCL10 release induced by cytomix. **Confluent serum-deprived ASMC were treated with ciglitazone and fluticasone or vehicle 1 h prior to and during stimulation with cytomix and CXCL10 release after 24 h quantified using ELISA.

In view of the inhibitory effects of ciglitazone on CXCL10 protein release, but not mRNA production, whether or not it was more effective when combined with fluticasone or salmeterol was also investigated. In non-asthmatic ASMC fluticasone 0.1-100 nM significantly reduced the inhibitory effects of 25 μM, but not 100 μM, ciglitazone on cytomix-induced CXCL10 release (Figure 7), whereas it had no significant effects in asthmatic ASMC. Salmeterol 0.1-100 nM caused no significant changes to any of the inhibitory effects of ciglitazone (data not shown).

None of the above treatments affected the viability of the ASMC.

## Discussion

In this study the inflammatory conditions under which current asthma therapies down-regulate production of the mast cell chemoattractant CXCL10 by ASMC from people with and without asthma were established. The GC fluticasone and LABA salmeterol very effectively inhibited CXCL10 release induced by TNF-α or IL-1β, whereas they were completely ineffective, individually or together, against IFNγ- or cytomix-induced CXCL10 release. Surprisingly, salmeterol at sub-nanomolar concentrations increased CXCL10 release induced by all three cytokines together (cytomix). This effect of salmeterol was most likely mediated by increased NF-κB activity, but does not appear to be a class effect, as low formoterol concentrations did not affect release. In addition, fluticasone did not prevent the salmeterol-induced CXCL10 increase. Importantly, the thiazolidinediones ciglitazone and rosiglitazone were effective inhibitors of cytokine-induced CXCL10 release. However, their action appeared to be independent of PPARγ and NF-κB p65 activity. Nevertheless thiazolidinediones may provide an alternative therapeutic strategy for GC and LABA resistant asthma.

TNF-α- or IL-1β-induced CXCL10 release by ASMC from people with and without asthma was strongly inhibited by GC and LABA treatment in this study. This is in agreement with previous studies in which fluticasone or salmeterol inhibited release of other asthma-relevant chemokines induced by TNF-α [10-12]. In addition, Clarke et al. [13] showed fluticasone had an inhibitory effect on TNF-α-induced CXCL10 release in non-asthmatic ASMC. Similarly, IL-1β induced chemokine production from lung epithelial cells was strongly inhibited by fluticasone, salmeterol or both combined [30]. Nie et al. [15] used ChIP analysis to demonstrate that the effects of fluticasone and salmeterol on ASMC TNF-α-induced eotaxin were mediated by inhibition of NF-κB p65 DNA binding to the eotaxin promoter. A similar mode of action could be assumed for TNF-α-induced CXCL10, as NF-κB also plays an important role in TNF-α-induced CXCL10 in ASMC [31].

In this study neither CXCL10 induced by IFNγ, nor CXCL10 induced by IFNγ combined with TNF-α and IL-1β (cytomix), were reduced when asthmatic and non-asthmatic ASMC were treated with GC or LABA individually, or in combination. In contrast, salmeterol has previously been reported to inhibit IFNγ–induced CXCL10 production by an airway epithelial cell line [32]. However, similar to this study, others observed the inhibitory effects of fluticasone on TNF-α-induced CXCL10 disappeared when non-asthmatic ASMC were stimulated with a combination of TNF-α and IFNγ [13,33]. Stimulation with TNF-α and IFNγ together inhibits ASMC glucocorticoid receptor (GR)α DNA binding and GC responsive element-dependent gene transcription [34,35]. The findings reported here may have clinical relevance because CXCL10 production in the airway mucosa of people with asthma is increased by oral corticosteroids [36].

Subnanomolar concentrations of salmeterol further up-regulated cytomix-induced CXCL10 secretion in asthmatic ASMC, whereas formoterol did not. Formoterol was used at the same concentrations on the same ASMC in parallel with salmeterol in the experiments and did not cause any changes in CXCL10 levels. Thus this effect of salmeterol was not a drug class effect, or due to differences in lung donor age. A difference in effects of the two LABA is perhaps not unexpected as these agents differ markedly in their agonist properties. Salmeterol does not associate with the β2-adrenoceptor preferentially as formoterol does. Instead it partitions to the outer lipid bilayer of cells where it is retained. Two models have been proposed for salmeterol receptor engagement: 1) it is anchored to an exosite and engages and disengages from the receptor, or 2) it diffuses through the bilayer to engage the receptor through the side [37,38]. In support of the latter, it has lower efficacy and onset of action than formoterol and increases membrane fluidity, which may affect receptor function and decrease its own efficacy reviewed in [39]. There is extensive evidence from the development of salmeterol that β2-adrenoceptor antagonists inhibit all its effects [40]. Given the reported efficacy of the β2-adrenoceptor antagonist butoxamine [27], its lack of effect on the salmeterol-induced increase in asthmatic ASMC CXCL10 release was unexpected. It is consistent with the effect of salmeterol not being mediated through classic β2-adrenoceptor engagement and signalling. However it does not eliminate the involvement of exosite binding or diffusion from the bilayer as salmeterol and butoxamine do not share all the same receptor binding sites [37,41]. It may be possible for salmeterol to bind to the exosite/receptor sites not blocked by butoxamine and activate signalling leading to the increase in CXCL10 production. Salmeterol ≥ 1pM increased IL-6 and CXCL8 production by the lung epithelial cell line BEAS-2B stimulated with IL-1β and histamine. The β2-adrenoceptor antagonist ICI 118551 blocked the effects of 100nM salmeterol, confirming β2-adrenoceptor involvement at that high concentration, but it was not tested against subnanomolar concentrations [42] similar to those used in this study.

NF-κB plays an important role in cytokine induced CXCL10 production. In intestinal epithelial cells, IL-1β and IFNγ alone, and in combination, up-regulated CXCL10 in an NF-κB dependent manner [43]. In ASMC from people with chronic obstructive pulmonary disease (COPD), TNF-α induced CXCL10 via NF-κB activation, whereas IFNγ only induced a weak activation of NF-κB [31] and activated STAT-1 as well, but not AP-1 [13]. Stimulation with these cytokines together results in synergistic increases in CXCL10 production by ASMC [9,13]. In this study, we showed in the asthmatic ASMC that cytomix stimulation strongly induced NF-κB p65 DNA binding activity and treatment with very low salmeterol concentrations further enhanced it. This increased p65 DNA binding activity may contribute to the increases in cytomix-induced CXCL10 and is consistent with the hypothesis that salmeterol increases asthmatic ASMC cytomix-induced CXCL10 production in an NF-κB dependent manner. However further studies are needed to establish whether or not the increase in p65 DNA binding activity is specific to asthmatic ASMC, occurs in the CXCL10 promoter and over what salmeterol concentration range this occurs.

The salmeterol enhancement of cytomix-induced CXCL10 release was specific to asthmatic ASMC. Whereas the salmeterol-induced increases in IL-6 and CXCL8 secretion by BEAS-2B cells were GC sensitive [42], the salmeterol enhancement of asthmatic ASMC CXCL10 production reported here was not. In ASMC from people with asthma there is increasing evidence of reduced sensitivity to steroids and intrinsic changes in intracellular signalling. These include altered calcium homeostasis and increased mitochondrial biogenesis [44,45]. Cytomix-induced NFκB transcriptional activity may be increased in the presence of salmeterol as a result because ASMC NFκB transcriptional activity is sensitive to changes in calcium [46]. As well, asthmatic ASMC β2-agonist–induced cAMP levels are lower due to increased PDE_4_ levels and activity [47] and cytomix-induced MAPK signalling is altered [9]. Production of cAMP activates cAMP-dependent protein kinase (PKA) and exchange protein directly activated by cAMP (Epac). Whether PKA and/or Epac expression or activity is altered in asthmatic ASMC warrants investigation. Notably, β2-agonists regulate non-asthmatic ASMC functions via Epac [48,49] and β2-agonist enhancement of bradykinin-induced CXCL8 release is mimicked by activation of either PKA or Epac and involves the MAPK ERK [49]. We reported recently that the MAPK JNK, but not p38 or ERK, is involved in CXCL10 production following cytomix stimulation, but JNK activation is markedly reduced in asthmatic compared to non-asthmatic ASMC [9]. Thus the salmeterol-induced increase in CXCL10 would seem less likely to be mediated via PKA or Epac with ERK. The alternate β-arrestin-2 pathway involved in receptor desensitization, leads to increased inflammation again through ERK/p38 MAPK activation reviewed in [50]. It may still be of interest however, as β-arrestin-2 also interacts with PI3K and some differences in expression of PI3K isoforms in asthmatic and non-asthmatic ASMC have been reported [51,52]. These scenarios are only likely if salmeterol does stimulate some receptor-mediated signalling in the asthmatic ASMC despite the presence of butoxamine. Clearly multiple signalling pathways are potentially involved and carefully controlled follow-up studies are needed to investigate which contribute to the salmeterol-induced increase in CXCL10 release by asthmatic ASMC and the functional consequences of this enhanced response.

The observed insensitivity of IFNγ- or cytomix-induced CXCL10 to current asthma medications emphasizes the need for novel compounds that can inhibit these nonresponsive pathways. In this respect thiazolidinediones show some promise. They are anti-inflammatory in animal models of asthma [20,21] and have bronchodilatory effects in smokers with asthma [53]. In vitro they do have anti-inflammatory and anti-remodelling effects, but these can be cell- and stimulus-specific and involve diverse and often complex mechanisms that are still being elucidated. Rosiglitazone, by increasing PPARγ signalling, inhibits CXC3CL1 signalling [54] and hypoxia-induced increases in lung TGF-b signalling and collagen deposition [55], but both pioglitazone and rosiglitazone reduce orbital fibroblast hyaluronan synthesis and T-cell adhesion to the fibroblasts in a PPAR-independent manner [56]. In ASMC they also have PPARγ-dependent and independent effects to inhibit ASMC proliferation and secretion of some key pro-inflammatory mediators including CCL11 (eotaxin), CCL2 (MCP-1), CCL5 (RANTES) and IL-6 [15,18,23].

In this study the thiazolidinedione ciglitazone strongly inhibited IFNγ- and cytomix-induced ASMC CXCL10 secretion. This appears to be a class effect as rosiglitazone, which is currently used to treat people with type II diabetes mellitus, also inhibited cytomix-induced CXCL10 release. Although Ward and colleagues [18] found the anti-proliferative effects of rosiglitazone in ASMC were sensitive to the PPARγ antagonist GW-9662, in this study GW-9662 did not prevent ciglitazone/rosiglitazone inhibition of asthmatic and non-asthmatic ASMC CXCL10 production. Further, in contrast to reports that PPARγ agonists can inhibit NF-κB activity [20] and subsequent CXCL10 production [57] in other cell types, there was no evidence that ASMC NF-κB activity was inhibited by ciglitazone or that it affected early CXCL10 gene transcription. In addition any reported thiazolidinedione effects on JAK-STAT signalling [58] are not applicable to inhibition of CXCL10 induced by IL-1β or TNF-α Thus we propose ciglitazone inhibits asthmatic and non-asthmatic ASMC CXCL10 release via a PPARγ-independent post-transcriptional mechanism. Nie and colleagues [15] have also provided evidence that PPARg agonists can have post-transcriptional inhibitory effects on ASMC chemokine production. They found the PPARγ agonist 15d-PGJ_2_ and thiazolidinedione troglitazone inhibited TNF-α-induced CCL2 post-transcriptionally, but prevented NFκB binding to the CCL11 gene promoter and inhibited CCL11 gene transcription. Zhu and colleagues [23] recently reported similar findings to our study: rosiglitazone and/or troglitazone inhibition of ASMC cytokine-induced IL-6 and CCL5 production was not prevented by GW-9662, or PPARγ knockdown, and was not accompanied by inhibition of NF-κB activation.

Although further studies elucidating the different mechanisms of action of the thiazolidinediones are needed, the strong inhibition of ASMC CXCL10 release observed, irrespective of cytokine stimulus, is evidence of another beneficial anti-inflammatory effect the thiazolidinediones might have in asthma and other obstructive respiratory diseases. However in contrast to the findings of Nie and colleagues for CCL5 and CCL11 [15], in this study there were no additional/synergistic inhibitory effects on CXCL10 release when ciglitazone was used in combination with fluticasone or salmeterol.

## Conclusions

It is widely accepted that the accumulation of activated mast cells within ASM bundles is important in the pathogenesis of eosinophilic asthma and that ASMC derived CXCL10 is involved in mast cell migration to the ASM [8]. In this study evidence of how current asthma therapies affect asthmatic ASMC CXCL10 release induced when different cytokines are present and the first evidence that salmeterol may sometimes increase CXCL10 release has been provided. As well, the efficacy of the thiazolidinediones in reducing asthmatic and non-asthmatic ASMC CXCL10 release has been demonstrated, even under inflammatory conditions where current asthma therapies were ineffective or enhanced release. Currently approved thiazolidinediones may be effective alternative treatments to reduce mast cell-ASM interactions and restore normal airway physiology in asthma.

## Abbreviations

ASM: Airway smooth muscle; ASMC: Airway smooth muscle cells; IκB-α: Nuclear factor of kappa light polypeptide gene enhancer in B-cells inhibitor, alpha; IL: Interleukin; TNF-α: Tumour necrosis factor-α; IFNγ: Interferonγ; NF-κB: Nuclear factor-κB; PPAR-γ: Peroxisome proliferator-activated receptor-γ; TGF-β: Transforming growth factor-β; GC: Gucocorticoids; LABA: Long-acting β2- agonists; Ig: Immunoglobulin; ChIP: Chromatin immunoprecipitation; STAT-1: Signal transducer and activator of transcription; AP-1: Activator protein 1; GR: Glucocorticoid receptor; cAMP: Cyclic adenosine monophosphate; PKA: cAMP-dependent protein kinase; Epac: Exchange protein directly activated by cAMP; MAPK: Mitogen activated protein kinase(s); ERK: p42/44 extracellular-signal-regulated kinase; JNK: Jun N-terminal kinase; PI3K: Phosphatidylinositol 3-kinase(s); JAK: Janus kinase(s); PG: Prostaglandin.

## Competing interests

The authors declare they have no competing interests.

## Authors' contributions

All authors contributed to the design of the study and the interpretation of the data. PS, HA and DJL conducted the experiments and analysed the data with the guidance of JMH. JMH, PS and HA prepared the manuscript. All authors read and approved the final manuscript.
